# Hypoglycemic and Anti-Inflammatory Effects of Triterpene Glycoside Fractions from *Aeculus hippocastanum* Seeds

**DOI:** 10.3390/molecules26133784

**Published:** 2021-06-22

**Authors:** Avez Sharipov, Khurshid Tursunov, Sunnatullo Fazliev, Bahtigul Azimova, Jamoliddin Razzokov

**Affiliations:** 1 Department of Inorganic, Physical and Colloidal Chemistry, Tashkent Pharmaceutical Institute, Oybek Street 45, Tashkent 100015, Uzbekistan; sharipov_a@pharmi.uz (A.S.); tursunov.khurshid9003@gmail.com (K.T.); baxt_gulim@rocketmail.com (B.A.); 2 Max Planck School Matter to Life, Jahnstrasse 29, 69120 Heidelberg, Germany; sunnatullo.fazliev@mtl.maxplanckschools.de; 3 Faculty of Chemistry and Earth Sciences, Heidelberg University, Im Neuenheimer Feld 234, 69120 Heidelberg, Germany; 4 Department of Physics and Chemistry, Tashkent Institute of Irrigation and Agricultural Mechanization Engineers, Kori Niyoziy 39, Tashkent 100000, Uzbekistan; 5 Department of Physics, National University of Uzbekistan, Tashkent 100174, Uzbekistan; 6 Institute of Material Sciences, Academy of Sciences, Chingiz Aytmatov 2b, Tashkent 100084, Uzbekistan

**Keywords:** chestnut, saponin, escin, biological activity, diabetes, inflammation

## Abstract

Horse chestnut (*Aesculus hippocastanum* L.)-derived drugs have shown their potential in biomedical applications. The seed of *A. hippocastanum* contains various kinds of chemical compounds including phenolics, flavonoids, coumarins, and triterpene saponins. Here, we investigated the chemical components in *A. hippocastanum* L. grown in Uzbekistan, which has not yet been studied in detail. We identified 30 kinds of triterpene saponins in an extract of *A. hippocastanum* L. Classifying extracted saponins into eight fractions, we next studied the hypoglycemic and the anti-inflammatory activities of escin and its derivatives through in vivo experiments. We came by data indicating the highest (SF-1 and SF-2) and the lowest (SF-5 and SF-8) antidiabetic and anti-inflammatory effects of those eight fractions. These results imply the prospective use of *A. hippocastanum* L. grown in Uzbekistan in the production of pharmaceutical drugs to treat diabetes and inflammation.

## 1. Introduction

In recent years, plant-derived extracts have gained a strong foothold in the fields of medicine, pharmacology, and food security [1]. The naturally extracted ingredients maintain their unique biological activities and can be used for various purposes [2]. For instance, the seeds of *Aesculus hippocastanum* L. (horse chestnut) are known to contain different complex compounds with potential therapeutic effects against diabetes, phlebitis, neuralgia, chronic venous and lymphatic insufficiency, diarrhea, fever, rheumatism, skin injuries, etc. [3,4,5,6,7]. In particular, a mixture of saponins, i.e., escin, present in *A. hippocastanum* L. was also evidenced to instantiate a substantial inhibitor of SARS viruses [8]. In addition, anticarcinogenic activity was also found in the isolated compound from the horse chestnut [9,10]. According to the investigations, approximately 20 individual compounds have been found in horse chestnuts, 70% of which contain escin, which is believed to indeed have higher therapeutic effects [10,11,12,13,14,15]. Initially, in 1960, Lorenz et al. extracted β-escin [16] including escin Ia, escin Ib, escin IIa, escin IIb, escin IIIa, isoescin Ia, isoescin Ib, isoescin IIa, isoescin IIb, deacetylescin Ia, deacetylescin Ib, deacetylescin IIa, and deacetylescin IIb. Moreover, there is also a record of research into the biological activity of escin V and VI [17,18,19,20].

Escin belongs to the triterpenoid saponin or glycoside group and is classified as α- and β-escin [21]. While the latter has been used as an antiseptic, antioxidant, analgesic, and antiaging compound [22], several experiments have pointed to new putative therapeutic activities of β-escin, namely, venotonic, anti-inflammatory [23,24], and antiedematous [16,25,26,27,28,29]. Disruption of proteolytic enzymes by escin results in activation of leukocytes, which is followed by decreased permeation of capillaries and veins, increasing the blood vessel wall’s tone [30]. The tension in veins accelerates the blood flow and, in turn, enhances microcirculation and delivery of oxygen to the tissues. Extensive in vivo and in vitro experiments, as well as a proposed mechanism of action of escins against inflammation and edema, can be found in the literature [25].

The genus *Aesculus* includes 12 species which belong to the Hippocastanaceae family. The abovementioned species are mainly found in Northern Hemisphere, specifically in territories of eastern Asia, southeastern Europe, and eastern America [3,31]. Moreover, horse chestnut is indigenous in the southern part of the Balkan peninsula, and the similar species are also widely cultivated in urban areas. The geographic location of Uzbekistan also offers a favorable habitat for horse chestnut. Chemical components in *A. hippocastanum* L. grown in Uzbekistan have not yet been studied in detail. Here, we present the isolation and characterization of derivatives of escin in *A. hippocastanum* L. native to the country. Our in vivo studies showed high hypoglycemic and anti-inflammatory activities of escin and its derivatives.

## 2. Results and Discussion

### 2.1. Identification of Chemical Structures of Saponin Derivatives by HPLC-MS

**Figure 1:**
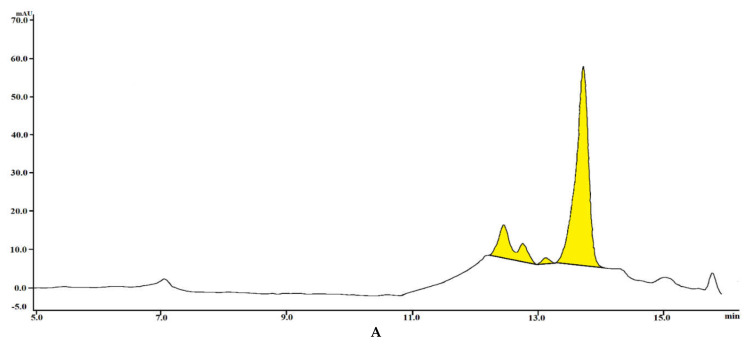

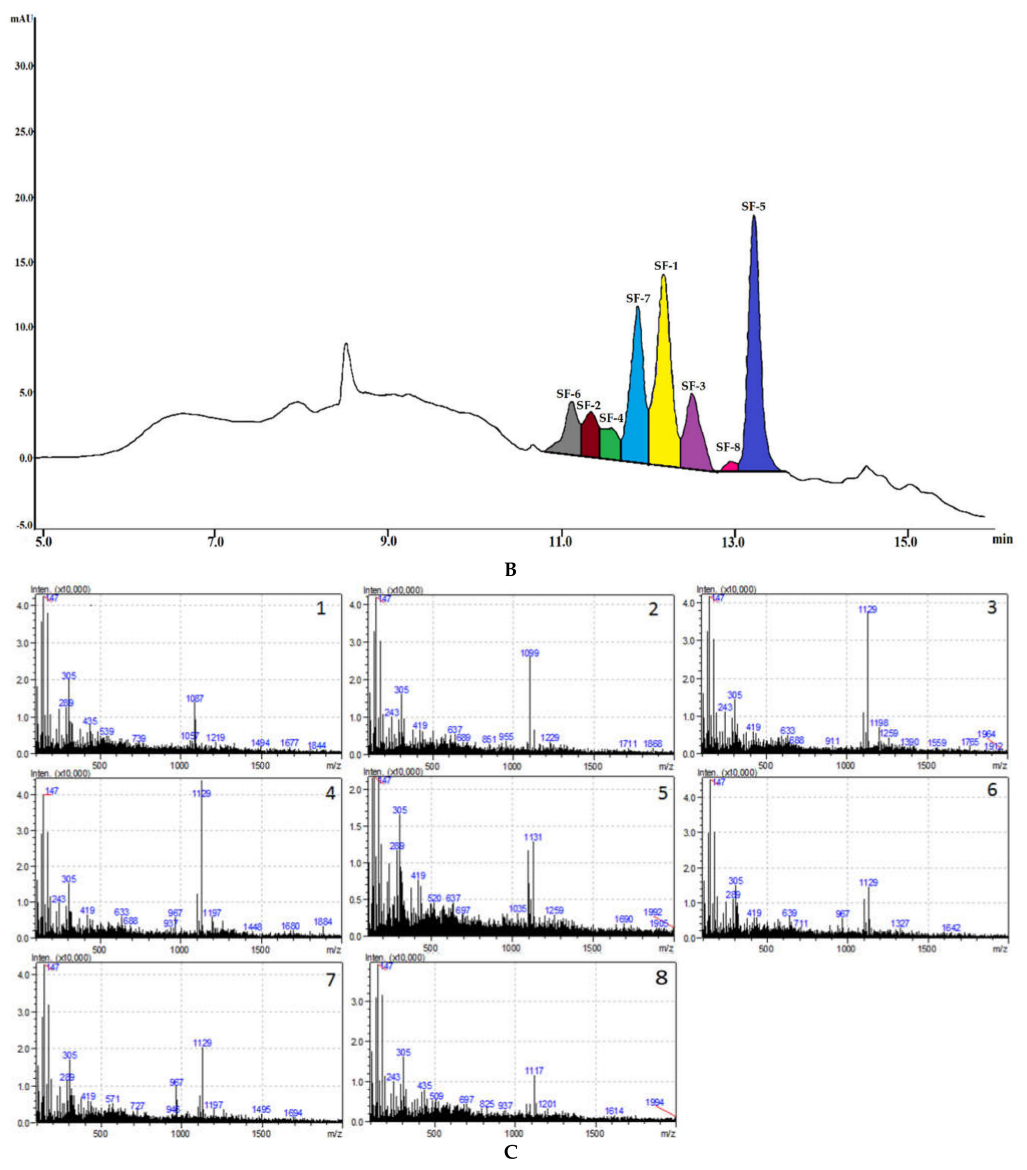
(**A**) Isolation and identification of saponins from the purified seeds of *A. hippocastanum L*. (**B**) Chromatograms of the standard escin and the extracts SF-1, SF-2, SF-3, SF-4, SF-5, SF-6, SF-7, and SF-8 from the seeds of *A. hippocastanum L*. (**C**) Chromatograms of the ES^‒^ purified from *A. hippocastanum L*. seeds. (**C**) The TICs for SF-1, SF-2, SF-3, SF-4, SF-5, SF-6, SF-7, and SF-8 (**1** for SF-1, **2** for SF-2, and so on). All chromatograms were obtained using a Shimadzu liquid system equipped with a single-quadrupole Shimadzu 2020 LC-MS system in negative electrospray ion mode with total diode array detection (DAD) at 226 nm.

Saponins derived from the seeds of *A. hippocastanum* L. and their derivatives were separated using preparative high-performance liquid chromatography (HPLC) and detected with single-ion monitoring mass spectrometry (SIM-MS) in negative ionic mode (Figure 1).

The natural chestnut seeds grown in Uzbekistan were analyzed (see Figure 1B) and found to contain 4.2% ± 1.3% triterpene saponins (see Figure 1A,B). The escin saponins (ES) were isolated (see Figure 1C) after gradual purification of the alcohol extract of chestnut seed (see Section 3.3). According to previous studies, multiple peaks occurred on escin’s chromatogram [19,32,33]. The main cause is that escins have more than 30 isomers and derivatives. Thus, even the chromatogram obtained by Kimura et al. using a standard sample of escin was shown to have a record of several peaks [34]. The peaks in the chromatogram of the sample seeds correspond to those of the standard sample of escin (see Figure 1A,B). Furthermore, analysis by ESI-MS showed that compounds with *m/z* [M − H]^−^ of 1129, 1099, 1131, 1113, 1089, 1117, 1087, and 1057 were detected (Figure 1C). In order to identify the chemical structure of escins, the obtained results were compared with the ^1^H-NMR and ^13^C-NMR data present in the literature [34].

**Figure 2:**
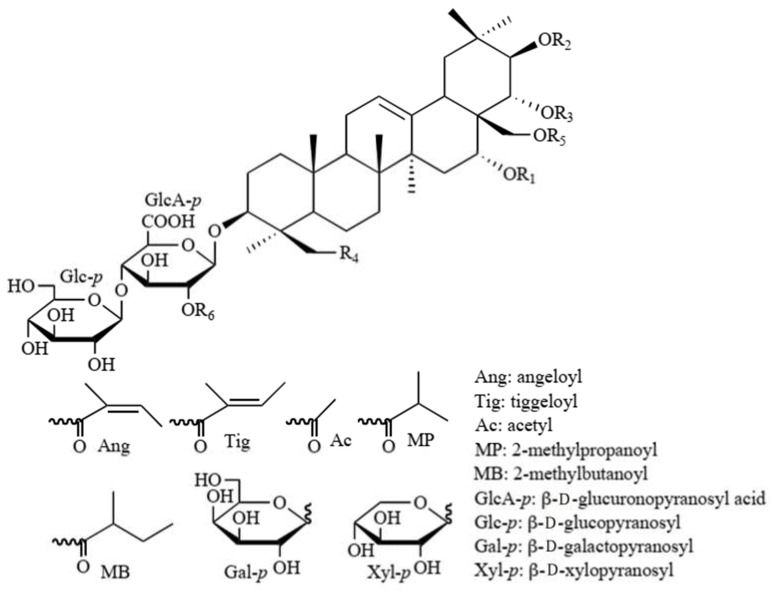
Structural formulas of escins.

Nine groups of peaks were identified on the basis of HPLC-MS data. The retention time of fake chestnut saponins (ES) ranged from 12.02 to 15.17 min (Figure 1C). The retention times of saponin fractions SF-1–8 were obtained by means of a total ion chromatogram (TIC) (Figure 1C). In our study, ES was divided into eight fractions according to their molecular weight and marked as saponin fraction (SFs) (see Figure 2 and Table 1).

**Figure 3:**
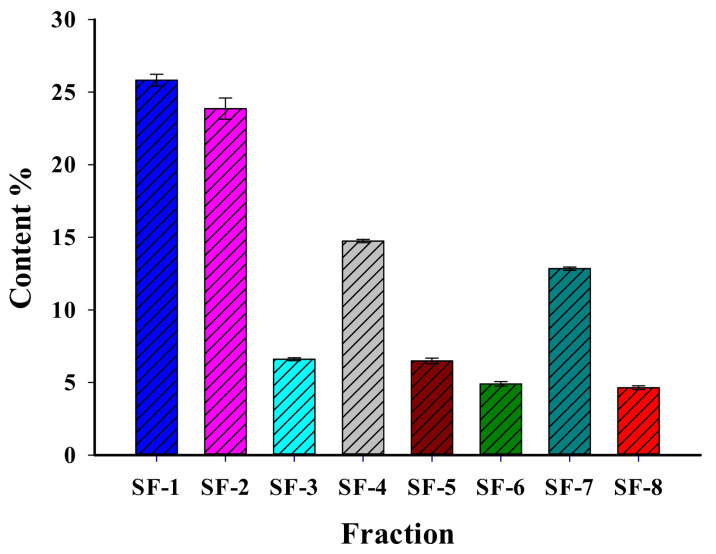
The proportion of saponin fractions in the isolated ES (*p* < 0.05).

We found that ES does contain 30 triterpene saponins (Table 1). We performed quantitative analysis of the eight fractions in the ES and determined the ratio of compounds as follows (see Figure 3): SF-1 (escin Ia, escin Ib, escin IVc, escin IVd, escin VIb, isoescin Ia, isoescin Ib, isoescin VIIa)—25.9%; SF-2 (escin IIa, escin IIb, isoescin IIa, isoescin IIb)—23.7%; SF-3 (escin IIIa, escin IIIb, isoescin IIIa, isoescin IIIb, isoescin VIIIa)—6.6%; SF-4 (escin IV)—14.7%; SF-5 (escin IVe, escin IVf, escin IVg, escin IVh, deacetylescin Ia, deacetylescin Ib)—6.5%; SF-6 (escin V, isoescin V)—4.9%; SF-7 (escin VI, isoescin VIa)—12.8%; SF-8, (deacetylescin IIa, deacetylescin IIb)—4.7%. These results show that approximately 50% of the ES consists of SF-1 and SF-2. In contrast, we found SF-8 to contain the lowest amount of escin.

Previous studies showed varying percentages of saponins present in fake chestnuts grown in different geographic locations. Yoshikawa et al. isolated saponins from the seeds of *A. hippocastanum* L. grown in Hiroshima (Japan). They divided extractions into six fractions and obtained the sum of escin Ia—0.141% (relative to raw materials), Ib—0.101%, IIa—0.080%, IIb—0.035%, IIIa—0.007%, and others [17,18]. We found the amount of saponins relative to the raw material to be 0.72% (SF-2), which is more than six times higher than that of chestnut seeds grown in Hiroshima (0.115%). Chen et al. detected saponins in *A. chinensis* seeds grown in Quingdao (China) by means of HPLC–MS. They obtained the sum of escin Ia—1.73% (relative to the raw material), escin Ib—1.04%, isoescin Ia—0.93%, isoescin Ib—0.59% (total—4.29%), and other escins divided into six fractions [35]. We identified that the amount of escin Is (SF-1, 1.17%) in *A. hippocastanum* L. seeds grown in Tashkent was 3.6 times lower than that found in *A. chinensis* seeds grown in Quingdao. It is obvious that the amount of saponins in the seeds of *A. hippocastanum* L. grown in the climate of Uzbekistan is different in comparison with other Asian countries.

### 2.2. Hypoglycemic Activity

In order to determine the nutrient activity of escin and its derivatives from the seeds of *A. hippocastanum* L., inhibition of the increase in blood glucose levels was tested by glucose tolerance tests in mice. The saponins were administered orally to two separate groups of mice at two different doses: 100 and 200 mg/kg. The 200 mg/kg dose of saponins was found to be effective in preventing an increase of blood glucose levels on mice after oral intake of glucose at intervals of 30 min and 1 h (see Table 2).

Furthermore, we examined the inhibitory effects of each SF against the increase in blood glucose and we obtained the following trend: SF-1 > (SF-1 + SF-2) > SF-2 > SF-6 > SF-3 > SF-4 > SF-7 > (SF-1 + SF-2 + SF-8) > SF-5 > SF-8. This observation showed that the tiggeloyl or angeloyl groups in C-21 and the acetyl moiety in C-22 are essential for the expression of high inhibitory activity toward elevated blood glucose levels.

The lowest hypoglycemic activity of SF-5 and SF-8 might be explained by the absence of the acetyl group in the structure of saponins present in these fractions. Saponins that do not contain the acetyl group in SF-8, when used in combination with those that contain acetyl group SF-1 and SF-2, resulted in an increase in hypoglycemic activity. This idea was also supported by a previous study on the inhibitory effect of escins isolated from Japanese fake chestnut seeds (*A. hippocastanum* L.) on elevated plasma glucose levels [34]. Thus, in hypoglycemia, the combined use of escins might be more effective. Certainly, the proposed hypothesis still requires more detailed research in order to establish clear conclusions.

### 2.3. Anti-Inflammatory Activity

**Figure 4:**
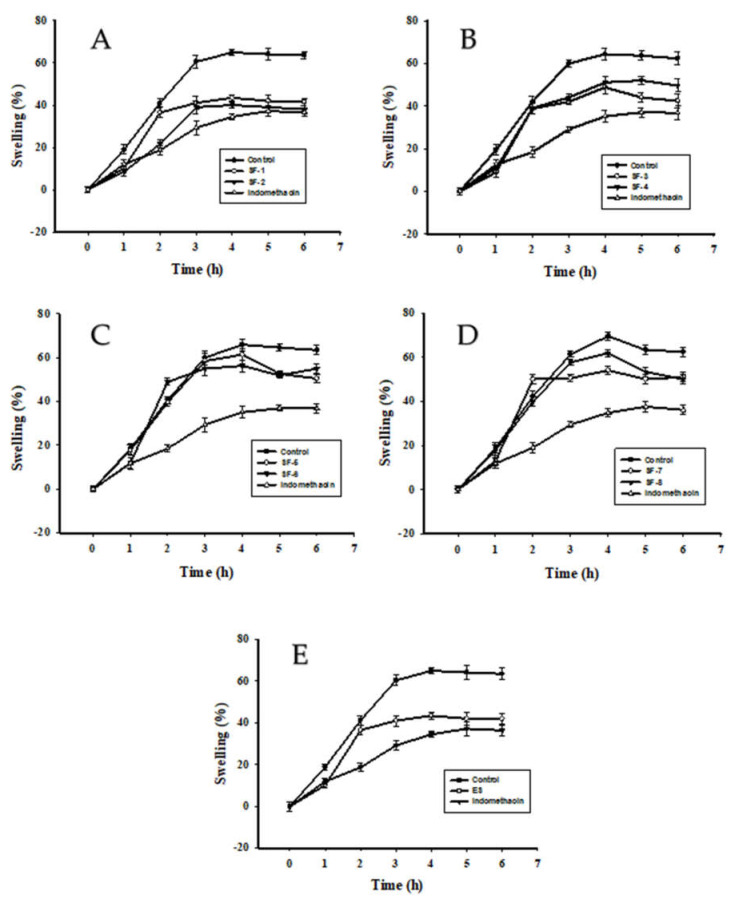
Anti-inflammatory effect of escins and their derivatives on inflammation induced by carrageenan in rat. (**A**)—SF-1, SF-2; (**B**)—SF-3, SF-4; (**C**)—SF-5, SF-6; (**D**)—SF-7, SF-8; (**E**)—ES. Statistically different from controls (*t*-test, *p* < 0.05). In fractions SF-3, SF-4 (**B**), SF-5, SF-6 (**C**), SF-7, SF-8 (**B**,**D**) and ES (**E**) we found a tendency toward inhibitory activity in the inflammatory models. From these results, we inferred that escin saponins isolated from the seeds of horse chesnut have an anti-inflammatory effect against inflammation caused by carrageenan. In addition, the presence of the acetyl moiety and the oligosaccharide probably plays an important role in anti-inflammatory activity. This can be seen in case of SF-5 and SF-8, which lack the acetyl moiety and showed reduced anti-inflammatory effects in our experiments.

Inflammation can be induced by applying various compounds such as lipopolysaccharides [36,37] and carrageenan [38,39,40]. We performed in vivo experiments using carrageenan as a mediator of inflammation. Furthermore, we investigated the anti-inflammatory activity of escin in acute inflammatory models. In rats, escin and its derivatives presented dose-dependent inhibitory effects in the early stages of development of edema induced by carrageenan. SF-1, SF-2 (see Figure 4A), and ED (Figure 4E) showed significant inhibition by carrageenan in the second stage of edema formation.

## 3. Materials and Methods

### 3.1. Materials

*A. hippocastanum* L. seeds were collected in the autumn of 2020 in the Gazalkent district of the Tashkent region (Uzbekistan). The seeds were air-dried (residual moisture 4–6%) and stored at room temperature until used.

An escin standard was used containing a 75% triterpenic glycoside pool (Sigma Aldrich, USA catalog No. PHL89871), sodium bicarbonate, ethyl alcohol, formic acid, acetonitrile for ultra-performance liquid chromatography (UHPLC; Gradient grade), and hydrochloric acid (Sigma Aldrich, Steinheim, Germany). Diaion HP-20 for column chromatography was obtained from Nippon Rensui (Tokyo, Japan). LC–MS analysis was carried out using a Shimadzu 2020 single-quadrupole LC–MS system, with an XR-ODS II 75 column (Shimadzu, Kyoto, Japan). All other chemicals were of reagent grade.

### 3.2. Preparation of Horse A. hippocastanum L. Seeds

The air-dried seeds of horse chestnut were peeled manually, and seed kernels were pulverized using a grinder to a size of 0.05–0.1 mm. Seed powder was defatted by hexane using a Soxhlet extractor. After removal of lipids, defatted seeds were dried under vacuum and kept at −20 °C before extraction.

### 3.3. Extraction, Fractionation, and Isolation of Saponins from Defatted A. hippocastanum L. Seeds

Preparation of plant extract. The preparation of plant extract was performed as described previously in the literature [41]. Briefly, the powdered seeds (2 kg) were extracted with 65% ethanol (EtOH) (2 L) by intermittent stirring at room temperature for 2 days, and the ethanol extract was filtered and the solvent was evaporated under reduced pressure below 40 °C. This process was repeated several times to remove the extractable components. The dry extract was dissolved in 200 mL of methanol. Next, this extract was transferred drop-by-drop to a flask of ice-cold diethyl ether stirring at a rate of 300 spin/min. The white precipitate was then filtered using a vacuum system through Whatman filter paper. The resulting precipitate was dried in a vacuum oven at 30–35 °C (yield: 46.15 g).

The ethanol extracts were applied to absorption column chromatography (500 mm × 60 mm i.d.) using Diaion HP-20 with 0.5 mm diameter particles (synthetic adsorbent). After washing the column with 3 L of distilled water to remove sugars, the fraction containing saponins (56 g of dried materials) was obtained by eluting with 3 L of ethanol.

To purify individual components of saponins from *A. hippocastanum* L. seeds, HPLC analysis was carried out on Shimadzu LC-2020 system equipped with a preparative HPLC column YMC-Pack ODS (150 mm × 10 mm i.d.). The preparative column was eluted at a flow rate of 3 mL/min with a mobile phase of methanol/10 mM sodium phosphate buffer (pH 7) (62:38, *v*/*v*). The elution of saponins was detected by monitoring the optical absorbance at 226 nm. For the analytical purposes, YMC-Pack ODS AM (150 mm × 6 mm i.d.) was eluted at a flow rate of 0.8 mL/min with the same mobile phase as above.

### 3.4. Instrumental Analysis

HPLC-MS analysis was carried out using the Shimadzu 2020 gas chromatograph–mass spectrometer with the XR-ODS II column (75 × 3 mm i.d.); the mobile phase was solution “A”—0.1% formic acid and solution “B”—acetonitrile. The pump program for gradient elution was as follows: start—10% “B”, 1—6 min, gradient “B” from 10 to 60% for 5 min; 6–8 min isocratic mode 60% “B”; 8–12 min linear gradient “B” from 60 to 95%; 12–15 min isocratic mode at 95% “B”; 15 min—reverse gradient “B” from 95 to 10% for 1 min; total analysis time 16 min. The chromatography profile was monitored using an SPD-M20A detector (200 to 400 nm), as well as the chromatogram of the total ion current. Mass spectra were recorded in scanning mode (electrospray ionization with registration of negative ions (ESI) with *m*/*z* values from 100 to 2000 (gas desiccant—nitrogen (flow 15 L/min), gas desiccant temperature—350 °C, flow rate of the nitrogen spray—1.5 L/min, ion source temperature—225 °C, voltage—14 V).

### 3.5. Animals

Sixty female and male Wistar rats (seven weeks old) and 55 female (4 weeks old) mice of the BALB/c strain were purchased from the Tashkent Pharmaceutical Institute. Animals (five per cage) were housed under standard conditions (light–dark cycle of 12 h, 20–22 °C room temperature, 50–60% humidity) and fed on a commercial diet and water ad libitum. Before the beginning of the experiment, animals were first acclimatized for 1 week to the lab conditions. All animals were used with the approval of the Ministry of Health of the Republic of Uzbekistan, Laboratory Animal Use Ethics Committee under the Veterinary Service. All procedures in this study were approved by the Tashkent Pharmaceutical Institute for animal experimentation and performed under isoflurane anesthesia.

### 3.6. Glucose Tolerance Test in Mice

After the mice were kept in food deprivation conditions for 16 h, the blood was withdrawn from the tail vein and subjected to the assay of blood level of glucose. Next, saponin fractions from natural seeds of chestnut were individually suspended in 0.3 mL of physiological saline. The resulting suspension was administered orally into the stomach of mice before a single oral injection of glucose (0.5 g/kg mouse) dissolved in 0.1 mL of physiological saline. Thereafter, the blood was withdrawn at 0.5, 1, and 2 h for analysis of blood glucose levels using GlucoDr Auto AGM-4000 (Gyeonggi-do, Republic of Korea) in compliance with the manufacturer’s instructions. The elevation of blood glucose levels was calculated by subtracting the blood glucose levels prior to the administration of glucose from those after the administration of glucose.

### 3.7. Experimental Design of the Study

All mice were randomly divided into eleven experimental groups and were treated as follows:

(i) Blank control group: injected with olive oil used as the vehicle (2 mL/kg);
(ii) SF-1-, SF-2-, SF-3-, SF-4-, SF-5-, SF-6-, SF-7-, and SF-8-treated groups: injected by a single oral administration of glucose at dose of 0.5 g/kg (*n* = 5);
(iii) Escin-treated groups: injected with two different doses (100 and 200 mg/kg) by a single oral administration of glucose at dose of 0.5 g/kg (per group *n* = 5).

#### 3.7.1. Evaluation of Anti-Inflammatory Activity

The male Wistar rats weighing 140–160 g were used for in vivo experiments. Inflammation was induced by carrageenin in these rats. Accordingly, 0.1 mL of 1% carrageenin was injected subcutaneously into the left hind paw l h after the administration of test samples [23]. The volume of the hind paw was measured by a plethysmometer (Animal research plethysmometer 37140, UgoBasile, Italy). The results were expressed as swelling (%), denoting the percentage increase in hind paw volume as compared with the initial volume.

All rats were randomly divided into 12 experimental groups after successful establishment of the carrageenan-induced inflammation model in rats (five rats per group), and they were injected orally according to their body weight for a single dose and treated as follows:

(i) Control group: carrageenin-induced inflammation rats received the vehicle of olive oil (2 ml/kg) by a single oral administration (*n* = 5);
(ii) SF-1-, SF-2-, SF-3-, SF-4-, SF-5-, SF-6-, SF-7-, and SF-8-treated groups: injected a dose of 100 mg/kg by a single oral administration (*n* = 5);
(iii) Escin-treated groups: injected with two different doses (100 and 200 mg/kg: carrageenin-induced inflammation + escin100 and carrageenin-induced inflammation + escin200) by a single oral administration (*n* = 5);
(iv) Carrageenin-induced inflammation rats received indomethacin 100 mg/kg by a single oral administration (*n* = 5).

#### 3.7.2. Carrageenan-Induced Acute Inflammatory Model

Anti-inflammatory activity was measured using the carrageenan-induced rat paw edema assay [41,42]. Edema was induced by subplantar injection of 100 μL of 1% freshly prepared solution of carrageenan in distilled water into the right hind paws of each rat of all the groups. Then, 30 min prior to carrageenan injection, paw thickness was determined before (0 h) and 1, 2, 3, 4, and 24 h after carrageenan injection. The increase in paw thickness was measured as the difference in paw thickness at 0 h and paw thickness at respective hours.

### 3.8. Statistical Analysis of Data

Data obtained from animal experiments were expressed as the mean ± standard error of mean (± SEM). Statistical differences between the treatments and control group were evaluated by one-way ANOVA. A difference in the mean values of *p* < 0.05 was considered to be significant.

## 4. Conclusions

We enhanced the extraction and isolation of saponins from the seeds of *A. hippocastanum* L. grown in Uzbekistan. Processing of the plant extract resulted in a pharmacological substance with more than 90% of its content being escin. An HPLC/MS analysis of the extracts showed that escin and its derivatives accounted for 4.2% ± 1.3% of the chemical components isolated from the seeds of *A. hippocastanum* L. Furthermore, we divided isolated saponin compounds into eight fractions on the basis of their molecular mass. and we investigated the antidiabetic and anti-inflammatory effects of the fractions in vivo. The results of the in vivo experiments indicated higher hypoglycemic and anti-inflammatory activity of the first and second fractions in comparison with the other six fractions. The absence of acetyl moiety and the oligosaccharide might have been responsible for the reduced biological activity of saponin fractions 3–8.

Escin saponins also show other potential pharmacological activities, for instance, against thromboembolism, viruses, and even cancer. Our future investigations will be focused on studying such new biological activities of escin saponins in a wider scope.

## Figures and Tables

**Table 1:** Identified escin saponins isolated from seeds of *A. hippocastanum* L.

1	RT (min)	Name	R_1_	R_2_	R_3_	R_4_	R_5_	R_6_	*m*/*z*; aem [M − H]^−^
SF-1	11.941	Escin Ia	H	Tig	Ac	OH	H	Glc-p	1129
		Escin Ib	H	Ang	Ac	OH	H	Glc-p	
		Escin IVc	H	H	Tig	OH	Ac	Glc-p	
		Escin IVd	H	H	Ang	OH	Ac	Glc-p	
		Escin VIb	Ang	Ac	H	OH	H	Glc-p	
		Isoescin Ia	H	Tig	H	OH	Ac	Glc-p	
		Isoescin Ib	H	Ang	H	OH	Ac	Glc-p	
		Isoescin VIIa	H	Tig	H	OH	Ac	Gal-p	
SF-2	10.791	Escin IIa	H	Tig	Ac	OH	H	Xyl-p	1099
		Escin IIb	H	Ang	Ac	OH	H	Xyl-p	
		Isoescin IIa	H	Tig	H	OH	Ac	Xyl-p	
		Isoescin IIb	H	Ang	H	OH	Ac	Xyl-p	
SF-3	11.343	Escin IIIa	H	Tig	Ac	H	H	Gal-p	1113
		Escin IIIb	H	Ang	Ac	H	H	Gal-p	
		Isoescin IIIa	H	Tig	H	H	Ac	Gal-p	
		Isoescin IIIb	H	Ang	H	H	Ac	Gal-p	
		Isoescin VIIIa	H	Ang	H	H	Ac	Glc-p	
SF-4	10.782	Escin IV	H	Ac	Ac	OH	H	Glc-p	1089
SF-5	13.210	Escin IVe	H	H	H	OH	Tig	Glc-p	1087
		Escin IVf	H	H	H	OH	Ang	Glc-p	
		Escin IVg	H	H	Tig	OH	H	Glc-p	
		Escin IVh	H	H	Ang	OH	H	Glc-p	
		Deacetylescin Ia	H	Tig	H	OH	H	Glc-p	
		Deacetylescin Ib	H	Ang	H	OH	H	Glc-p	
SF-6	10.343	Escin V	H	MP	Ac	OH	H	Glc-p	1117
		Isoescin V	H	MP	H	OH	Ac	Glc-p	
SF-7	10.938	Escin VI	H	MB	Ac	OH	H	Glc-p	1131
		Isoescin VIa	H	MB	H	OH	Ac	Glc-p	
SF-8	12.587	Deacetylescin IIa	H	Tig	H	OH	H	Xyl-p	1057
		Deacetylescin IIb	H	Ang	H	OH	H	Xyl-p	

**Table 2:** Results of the oral glucose tolerance test to monitor inhibitory effects of escins and their derivatives on the elevation of blood glucose levels in mice.

Sample	Dose (mg/kg, p.o.)	Elevation of Blood Glucose Level (mg/dL)		
		0.5 h	1.0 h	2.0 h
Control	0	112.9 ± 19.71 (100)	82.4 ± 13.26	51.2 ± 11.38
Ecsin and its derivatives (ED)	200	47.2 ± 12.69 (41.8) **	44.0 ± 8.85	48.2 ± 9.49
SF-1	100	67.4 ± 13.46 (59.7) *	70.88 ± 16.65	52.5 ± 10.86
SF-2	100	76.9 ± 8.97 (68.1) **	85.9 ± 11.87	51.9 ± 9.33
SF-3	100	84.8 ± 11.9 (75.1) *	92.8 ± 9.62	52.3 ± 7.01
Control	0	111.7 ± 15.0 (100)	68.9 ± 12.41	48.8 ± 8.20
Ecsin and its derivatives (ED)	100	86.4 ± 12.1 (77.4) *	69.8 ± 12.25	50.8 ± 8.42
SF-4	100	84.3 ± 11.1 (75.5) **	56.6 ± 8.09	48.9 ± 7.91
SF-5	100	92.2 ± 11.5 (82.5) *	57.5 ± 8.32	51.3 ± 8.43
SF-6	100	81.9 ± 15.0 (73.3) *	62.8 ± 6.86	49.7 ± 8.66
Control	0	114.7 ± 14.8 (100)	72.2 ± 16.26	58.5 ± 7.47
SF-7	100	89.2 ± 10.7 (77.8) **	76.6 ± 16.91	61.7 ± 7.22
SF-8	100	109.9 ± 13.2 (95.8) *	75.5 ± 12.84	62.9 ± 8.41
SF-1 + SF-2	100	74.8 ± 9.0 (65.2) *	66.1 ± 8.76	57.8 ± 12.00
SF-1 + SF-2 + SF-8	100	86.2 ± 7.5 (75.2) *	63.0 ± 14.83	51.7 ± 8.95

Each sample was orally administered to mice 30 min before oral administration of d-glucose (0.5 g/kg). Values in parenthesis show the percentage difference in plasma glucose concentration between control and each sample treatment. Data represent the mean ± SEM (*n* = 5). * *p* < 0.05, ** *p* < 0.01.

## Data Availability

Data is contained within the article.

## Sample Availability

Samples of the compounds are available from the authors.
